# A novel all-in-one strategy for purification and immobilization of β-1,3-xylanase directly from cell lysate as active and recyclable nanobiocatalyst

**DOI:** 10.1186/s12934-021-01530-5

**Published:** 2021-02-06

**Authors:** Lixi Cai, Yunmen Chu, Xin Liu, Yue Qiu, Zhongqi Ge, Guangya Zhang

**Affiliations:** 1 Department of Bioengineering and Biotechnology, Huaqiao University, Xiamen, 361021 Fujian China; 2 Faculty of Basic Medicine, Putian University, Putian, 351100 Fujian China

**Keywords:** β-1,3-xylanase, Enzyme immobilization, Silica nanoparticles, SpyCatcher, SpyTag

## Abstract

**Background:**

Exploring a simple and versatile technique for direct immobilization of target enzymes from cell lysate without prior purification is urgently needed. Thus, a novel all-in-one strategy for purification and immobilization of β-1,3-xylanase was proposed, the target enzymes were covalently immobilized on silica nanoparticles via elastin-like polypeptides (ELPs)-based biomimetic silicification and SpyTag/SpyCatcher spontaneous reaction. Thus, the functional carriers that did not require the time-consuming surface modification step were quickly and efficiently prepared. These carriers could specifically immobilize the SpyTag-fused target enzymes from the cell lysate without pre-purification.

**Results:**

The ELPs-SpyCatcher hardly leaked from the carriers (0.5%), and the immobilization yield of enzyme was up to 96%. Immobilized enzyme retained 85.6% of the initial activity and showed 88.6% of the activity recovery. Compared with free ones, the immobilized β-1,3-xylanase showed improved thermal stability, elevated storage stability and good pH tolerance. It also retained more than 70.6% of initial activity after 12 reaction cycles, demonstrating its excellent reusability.

**Conclusions:**

The results clearly highlighted the effectiveness of the novel enzyme immobilization method proposed here due to the improvement of overall performance of immobilized enzyme in respect to free form for the hydrolysis of macromolecular substrates. Thus, it may have great potential in the conversion of algae biomass as well as other related fields.

## Background

Marine algae, one of the most low-cost feedstock candidates for biofuels and chemical production, contribute approximately 50% of the earth’s primary production and generate huge numbers of distinctive polysaccharides that didn’t exist in land plants [1–3]. As a homopolysaccharide composed of β-1,3-linked d-xylose units, β-1,3-xylan exists in the cell walls of some red and green algae instead of cellulose [4–6]. β-1,3-xylanase (EC3.2.1.32) could cleave β-1,3-xylosidic linkages to produce multifarious xylooligosaccharides, which play an important role in bioenergy production and the renewable chemical commodities from marine algae [7–9]. However, the difficultly to separate the free β-1,3-xylanases from the hydrolyzed β-1,3-xylan solution and the low reuse rate became the bottlenecks of algae biomass conversion [10]. Accordingly, the strategy of enzyme immobilization provides a useful means to circumvent these problems by saving the usage of enzyme, simplifying downstream processing and also improving operational stability [11, 12].

Moreover, enzyme purification is the well-known obstacles in developing cost-effective processes to produce the immobilized enzyme. Thus, exploring a simple and versatile technique for direct immobilization of targeted enzymes from cell lysate without prior purification steps is a desirable strategy [13]. SpyCatcher is a peptide that spontaneously forms an isopeptide bond with its partner SpyTag. The reaction could occur rapidly under mild conditions with high efficiency and specificity, and the covalent bond between them is robust to diverse harsh conditions [14–17]. Thus, SpyTag/SpyCatcher is deemed a novel and efficient molecular adhesion that plays a vital role in the immobilization of enzymes [18–20].

Elastin-like polypeptides (ELPs) are known as a kind of thermally-responsive polypeptides with repeat pentapeptide motifs of Val-Pro-Gly-Xaa-Gly (where Xaa can be any amino acid other than proline) [21, 22]. As a purification tag, ELPs-based protein purification process is large-scalable, timesaving and cost-effective [23–25]. Recently, the cationic ELPs [KV8F-40] (where 40 repeats of the pentapeptide motifs) were demonstrated to have the capability of rapidly prepared biomimetic silica nanoparticles (NPs). The specific mineralization activity was positively correlated with the content of basic amino acids (such Lys) [26].

In this paper, we designed a novel ELPs [K5V4F-40] (where K5V4F means the ratio of K:V:F = 5:4:1, named K5 according) and with the *p*I value of 9.64. Then, the ELP was fused with SpyCatcher (named K5-C). The K5-C still had the capability of preparation of biomimetic silica NPs with the SpyCatcher on the surface of silica NPs. On the other hand, the target β-1,3-xylanase fused with a SpyTag was successfully expressed and the cells of host strains were ruptured by sonicator. Then the K5-C modified silica NPs (K5-C@SiO_2_) could spontaneously forms a covalent bond with SpyTag in the target β-1,3-xylanase (Additional file 1: Fig. S1). Thus, an all-in-one strategy for purification and immobilization of the target enzymes was proposed, which would pave a new way for the preparation of covalently immobilized enzymes for research or commercial purpose.

## Results and discussion

### Expression and purification of the K5-C and β-1,3-xylanases

**Fig. 1:**
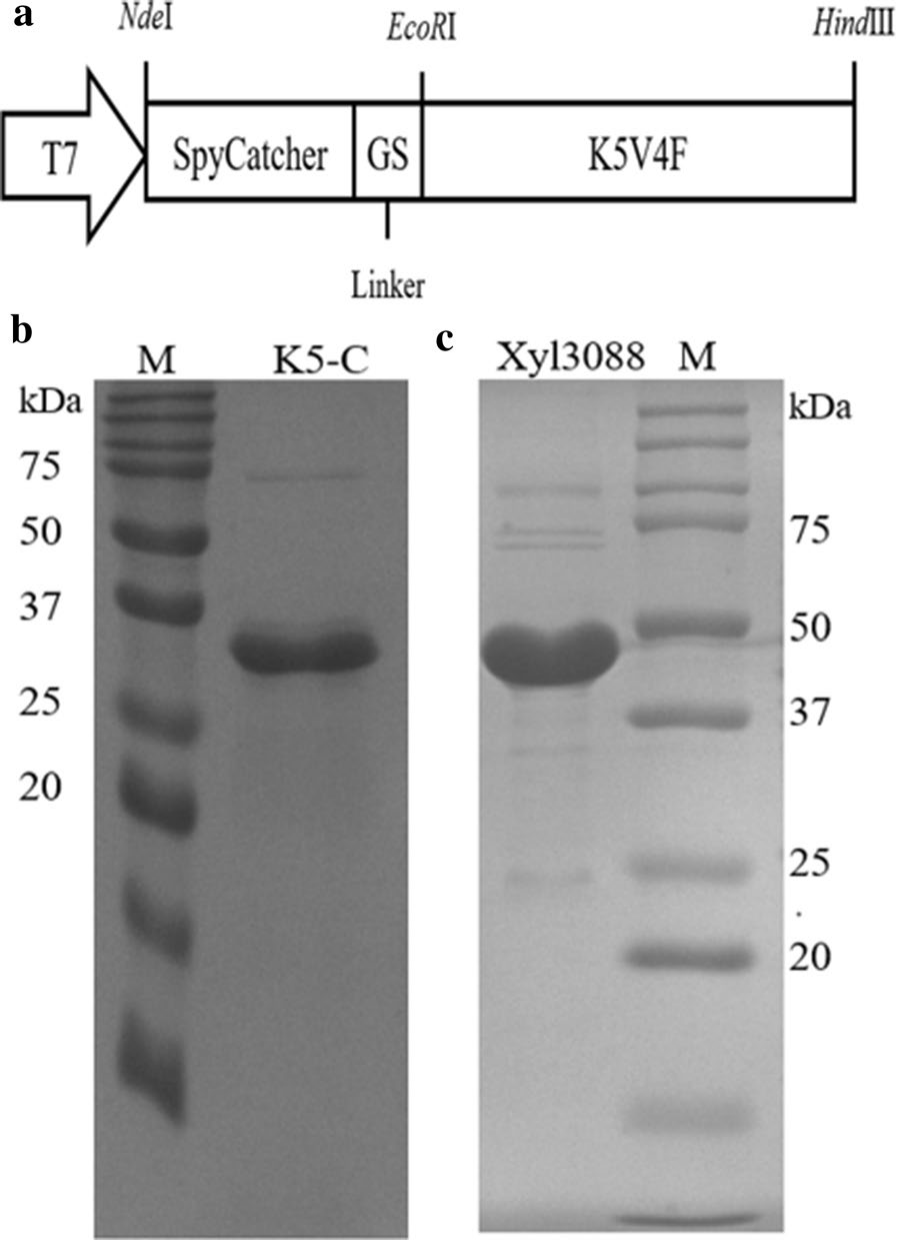
**a** Gene construction of K5-C. **b** The SDS-PAGE of purified K5-C. **c** The SDS-PAGE of purified β-1,3-xylanases (Xyl3088)

The diagrammatic sketch of the K5-C chimera was shown in Fig. 1a. The gene coding the SpyCatcher was fused to the K5V4F gene provided by the parent vector. After expressed in the host strain and purified, the purity and molecular weight of K5-C and β-1,3-xylanases (Xyl3088) were characterized by SDS-PAGE. For the purity of K5-C and Xyl3088, SDS-PAGE yielded a clear thick band of 32 kDa and 49 kDa, respectively (Fig. 1b, c), which matched their theoretical molecular weight values of 32002 Da and 48853 Da calculated by ProtParam (http://web.expasy.org/protparam/). The final yield of K5-C and Xyl3088 measured by BCA protein assay was 273.05 ± 11.5 mg and 13.65 ± 1.4 mg from 1 L culture, respectively. It means that the large-scale preparation of K5-C protein can be easily achieved by two rounds of ITC which requires only inexpensive reagents such as sodium chloride and simple centrifugation techniques [21, 27]. Thus, the expensive chromatographic purification processes were avoided. In addition, the K5-C has the ability of biomimetic silicification and can be used for low-cost mass production. It can be used in subsequent large-scale preparation of nanoparticles for immobilizing the targeted enzymes [22, 28]. Finally, the free β-1,3-xylanases was purified by nickel column and the quantity-calculating results revealed the Xyl3088 comprised about 92% of the total soluble proteins after purification, which can be used for further investigation into the characteristics.

### Synthesis of silica nanocomposites by K5C

**Fig. 2:**
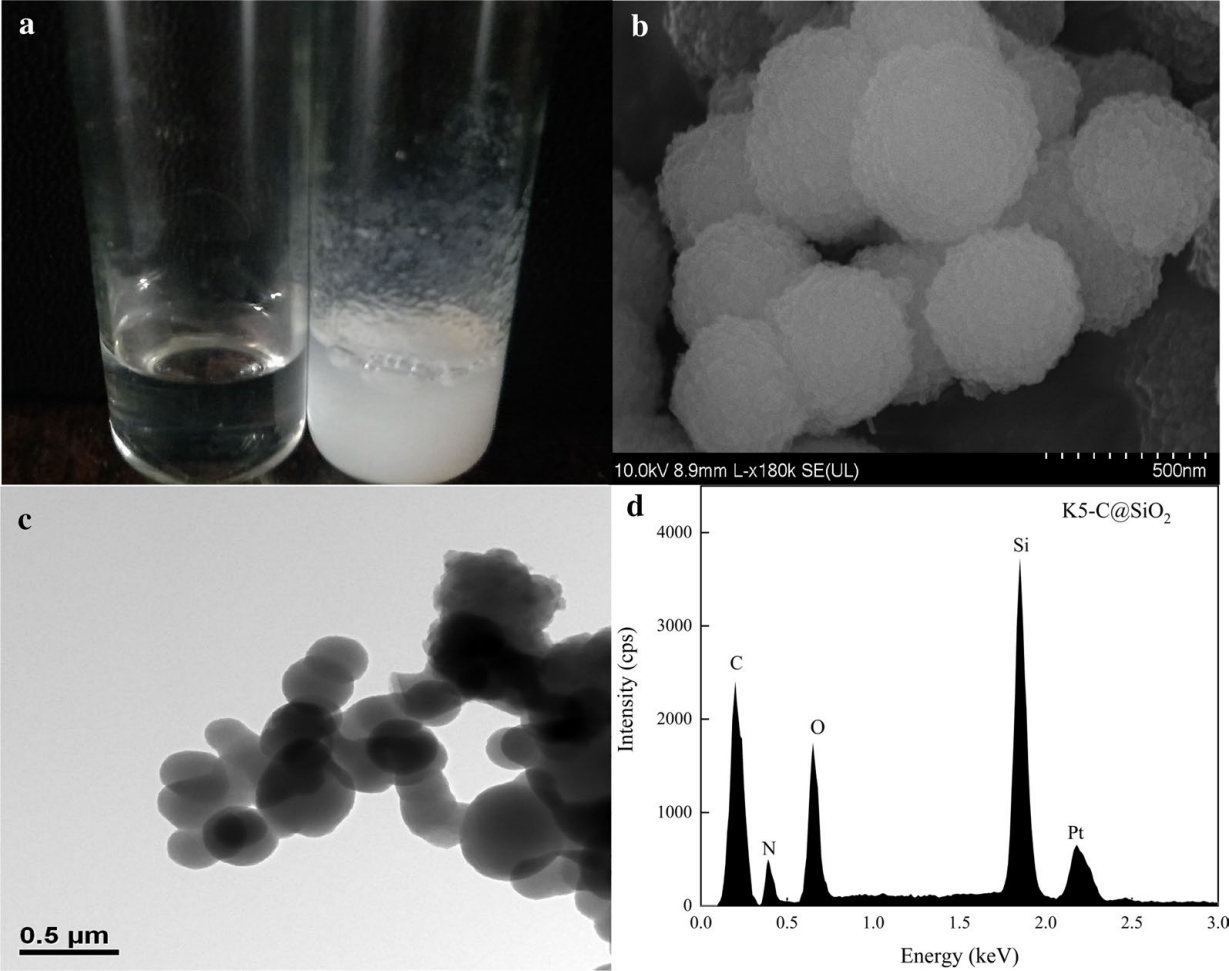
**a** Silica formation in TMOS without (left) and with (right) K5-C protein. **b** SEM micrograph of K5-C@silica (scale bar is 0.5 μm). **c** TEM micrograph of K5-C@silica (scale bar is 0.5 μm). **d** EDS analysis of K5-C@silica

When freshly prepared TMOS was loaded to K5-C solution under room temperature, white precipitation was mediated within seconds in PBS solution (Fig. 2a, right). While, no precipitation was observed in the negative control using the same reaction system without K5-C (Fig. 2a, left), indicating that K5-C is a vital template for silica synthesis. The scanning electron microscopy (SEM) images showed that the white precipitation mediated by K5-C were spherical with rough **s**urfaces, and the diameters were from 200 to 600 nm (Fig. 2b). This suggested that high surface area for the anchoring of numerous SpyCatcher, which was beneficial to immobilize enzymes by covalent bond. The transmission electron microscope (TEM) images further confirmed the morphology and size of the white precipitation (Fig. 2c). Besides, strong signals for C, O, N and Si in the white silica nanosphere were analyzed by energy dispersive spectrometer (EDS) experiment (Fig. 2d), indicating that K5-C fusion proteins were successfully encapsulated in the silica nanospheres (NPs). Hence, the silica NPs were organic–inorganic hybrid complexes, which can be applied for subsequent target enzyme immobilization.

### Influences of K5-C concentration on the immobilization efficiency

**Fig. 3:**
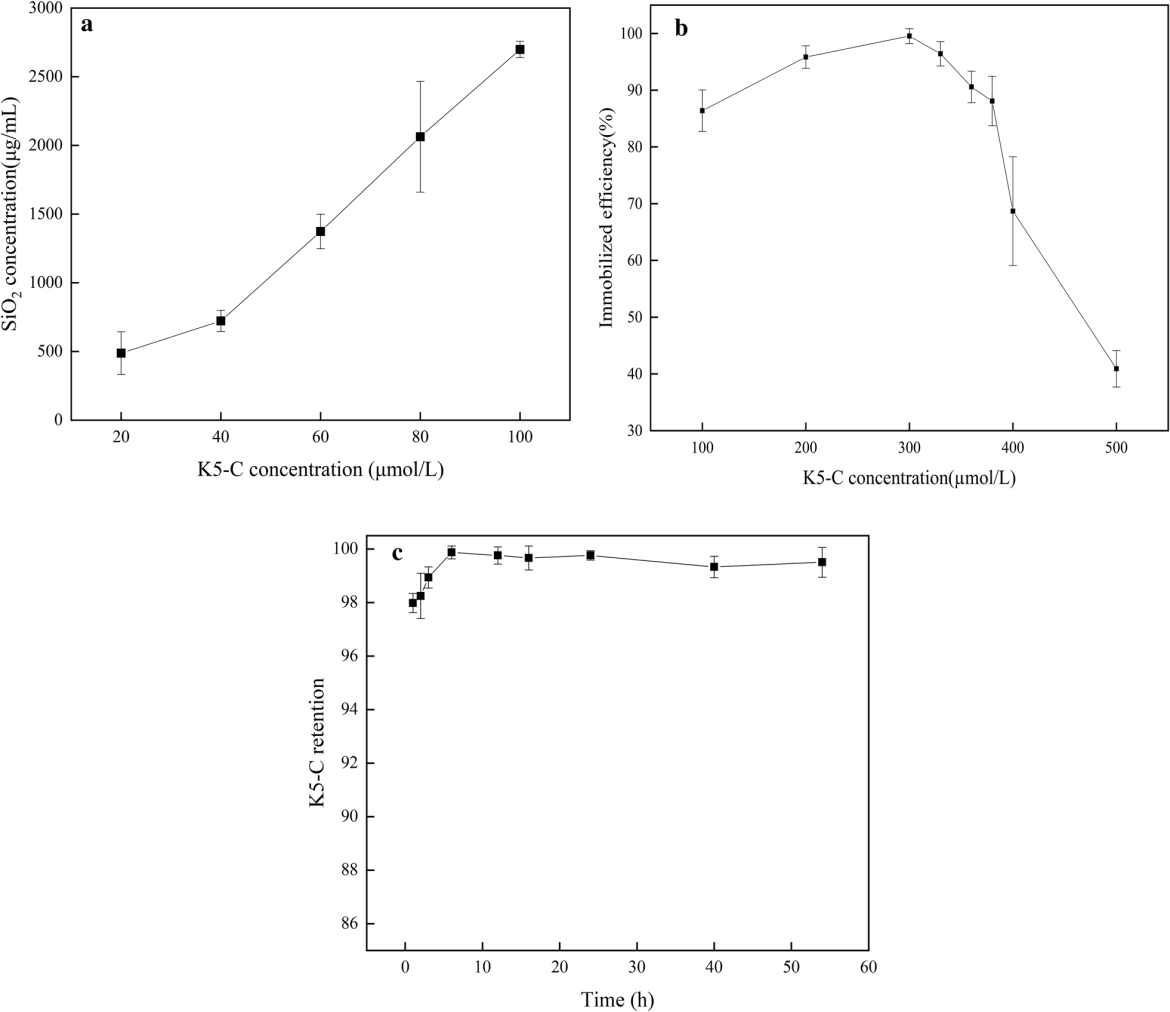
**a** Correlation between K5-C concentration and the amount of silica precipitated from TMOS. **b** K5-C immobilization efficiency at various concentrations. **c** Leaching test of K5-C embedded in silica supports according to the incubation time

To quantitatively analyze the mineralization activity of K5-C, the correlation between K5-C concentration and the yield of silica NPs was measured. As shown in Fig. 3a, the yield of silica was almost proportional to the amount of K5-C applied under premise controlling of the amount of silica precursor (TMOS). It was also consistent with some previous studies which silaffin, ELP(KV8F) and R5 were used to form silica nanospheres [28–30]. When the concentration of K5-C was 100 μmol/L, 44.97% of TMOS was converted to silica NPs. Meanwhile, the specific activity of K5-C, R5 and ELP(KV8F) was listed in Additional file 1: Table S1. Under the same reaction conditions, the specific activities of silaffin and R5 were only between 2 and 4, while the activities of K5-C and KV8F reached 99.93 and 97.18, respectively. These results proved that K5-C was a silica mineralized polypeptide with high catalytic activity and the introduction of lysine enhanced the activity of the polypeptides.

To optimize K5-C immobilization efficiency, the concentration of K5-C varied from 100 to 500 μmol/L. As can be seen in Fig. 3b, higher ratios of K5-C could self-immobilize in silica NPs with the increasing concentrations of K5-C. Approximately 99% of K5-C protein were immobilized when the concentration was 300 μmol/L, and then the immobilization efficiency decreased with the increase of K5-C concentrations. This may be due to the exhaustion of TMOS, resulting in the excess of K5-C. It means that K5-C could almost self-immobilized in the silica NPs by completely consuming TMOS at 300 μmol/L.

The capacity of immobilized K5-C for applications was further investigated by studying the extent of leaching [31]. As shown in Fig. 3c, the silica NPs could firmly entrap the K5-C protein and negligible protein leakage was observed in the supernatant of silica NPs even after 56 h storage at 4 °C, indicating that K5-C was strongly embedded inside the silica NPs, while the amine-based biosilica only retain 90% of the enzyme after 24 h of storage [31, 32]. Accordingly, K5-C@SiO_2_ NPs was a stable carrier displaying SpyCatcher on NPs surface, resulting in efficient assembly of the target enzymes in subsequent steps.

### Purification and immobilization of β-1,3-xylanases in one-step

**Fig. 4:**
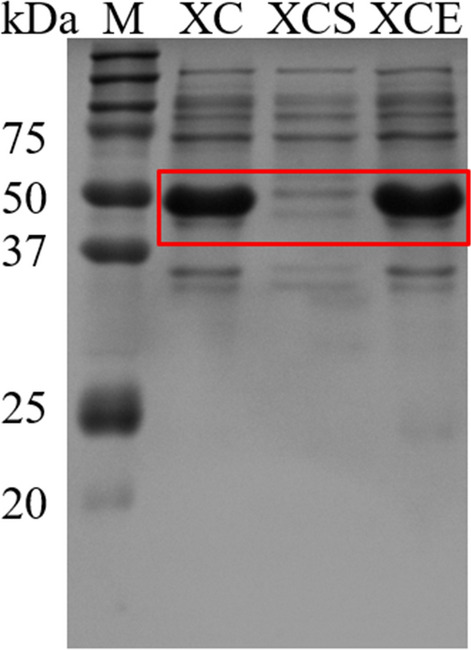
Analysis of X-T immobilization and purification by the SDS-PAGE. Red box revealed the position of the bands corresponding to X-T. Lane: M: molecular weight marker; XC: X-T cell lysate; XCS: the supernatant of X-T cell lysate after immobilization employing K5-C@SiO_2_. XCE: the supernatant of X-T cell lysate after incubating with K5@SiO_2_

To date, most of the enzyme immobilization techniques required prior purification of the target enzymes [33–35], which was a well-known process of costly and time-consuming [36]. Hence, development of a simple and versatile strategy for simultaneous purification and immobilization of the target enzymes was highly desirable [13]. Here, the silica NPs containing SpyCatcher on the surface as a carrier was loaded in the cell lysate of the chimeras of SpyTag fused β-1,3-xylanases (X-T). The immobilization and purification results were shown in Fig. 4, the supernatant of X-T cell lysate after employing K5-C@SiO_2_ revealed the loss of X-T band (51 kDa), while the amount and location of other impurities remained unchanged (Fig. 4, 2nd lane). Meanwhile, the loading of K5@silica NPs without SpyCatcher did not change the targeted X-T and other impurities (Fig. 4, 3rd lane), especially the target X-T. Accordingly, it clearly revealed that K5-C@SiO_2_ can specifically and covalently react with the X-T (via SpyCatcher and SpyTag) from the cell lysate in one-step. The immobilization efficiency and activity recovery of the captured β-1,3-xylanases (xyl3088) reached 85.4% and 88.6%, respectively.

Self-immobilizing and purification systems possessing such tags offer advantages since they immobilize targeted enzymes under mild and non-toxic environment while retaining stereoselectivity and activity [37]. Besides, they would also save costs due to avoidance of crosslinkers and simplification of immobilization processes, such as purification and immobilization directly from crude cell lysate without any prior costly purification steps [38].

### Effect of temperature and pH on the activity of the free and immobilized β-1,3-xylanases

**Fig. 5:**
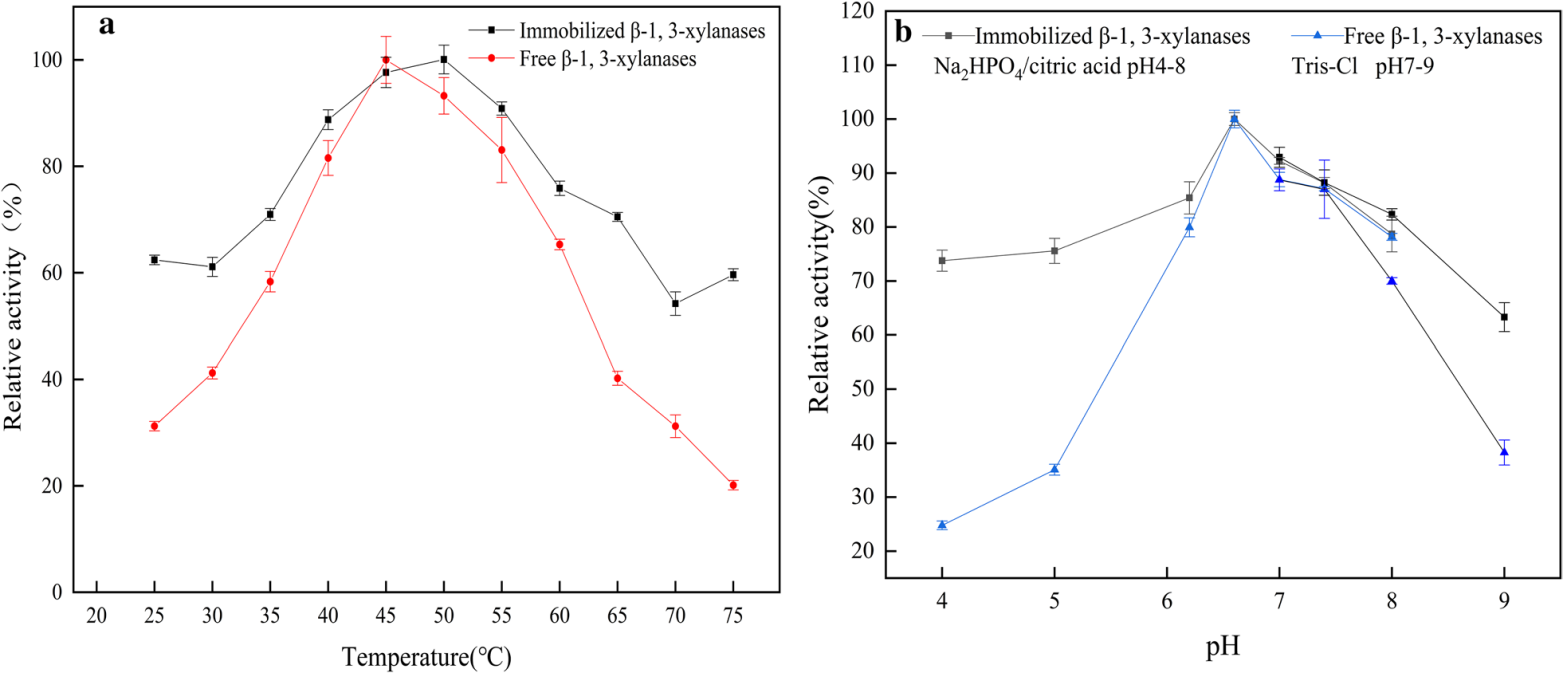
**a** Effect of temperature on the activity of β-1,3-xylanases. **b** Effect of pH on the activity of β-1,3-xylanases

The ability of an enzyme may be modulated by its immediate microenvironment [39]. So, the activities of free and immobilized Xyl3088 were assayed at temperatures ranging from 25 to 75 °C (Fig. 5a). The relative activities of immobilized β-1,3-xylanases (Xyl3088) were higher than those of the free ones over most of the temperature points, indicating that the immobilized Xyl3088 had more preferable temperature adaptability than the free one. The maximum activity of the free Xyl3088 was observed at 45 °C, while the optimum temperature of the immobilized Xyl3088 shifted up to 50 °C. The rise in optimum temperature may be owe to the reducing conformational flexibility, which requires a higher activation energy for the molecule to reorganize its proper conformation to bind to the substrate [40], thus activity even at higher temperature is one of the main advantages of immobilized enzymes [41].

The optimum pH for the activity of an enzyme was mainly dependent upon the nature of its functional groups. Besides, binding enzyme on a solid matrix may increase the pH tolerance depending on the surface and residual charges of the solid matrix [42, 43]. As shown in Fig. 5b, the optimum pH of both the free and immobilized Xyl3088 were observed at 6.6. But the immobilized Xyl3088 was found to be more stable than the free Xyl3088 from pH 4.0 to 9.0, this may be due to an increase in net charge arising from the binding of the enzyme to the silica NPs [44].

### The thermal stability, storage stability and reusability of the free and immobilized β-1,3-xylanases

**Fig. 6:**
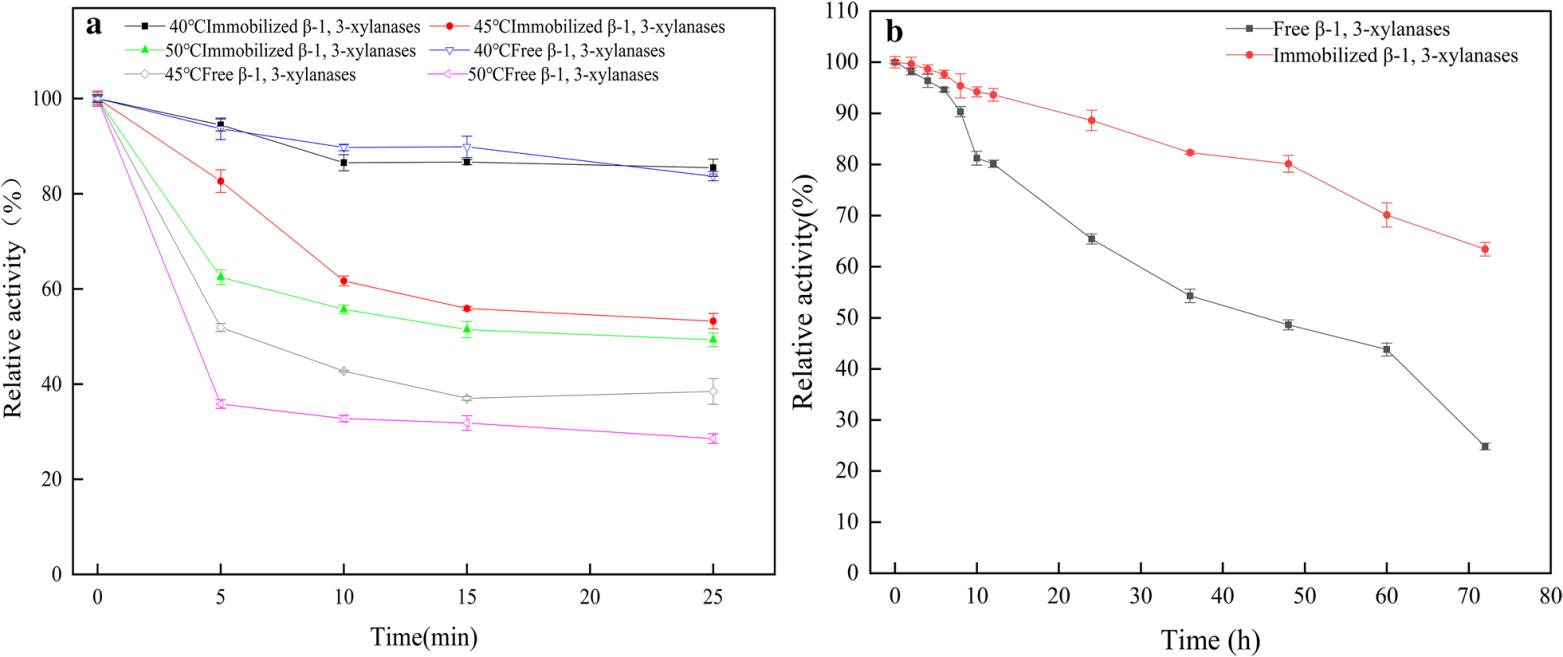
**a** Thermal stability of the free and immobilized Xyl3088. **b** Storage stability of the free and immobilized Xyl3088 at 30 °C

The activity of β-1,3-xylanases (Xyl3088) was highly sensitive to temperature, therefore, improving the thermostability of them is very important for potential industrial application. The thermostability of the free and immobilized Xyl3088 were shown in Fig. 6a, the immobilized Xyl3088 was more stable than the free one after incubation at 40 °C, 45 °C and 50 °C, respectively. This was further confirmed by the half-life test (Table 1). For example, the half-life of the free Xyl3088 reduced significantly in respect to the immobilized form (the half-life of the immobilized Xyl3088 in PBS buffer at 50 °C was about 28 min, which was 1.65-fold longer than the free one). The t-test result showed the *p* value was less than 0.01, revealing the differences between them were extremely significant. It also indicated that covalent immobilization might change the conformation of β-1,3-xylanases, resulting in higher thermostability towards temperature compared with the free ones [45–47]. Meanwhile, the storage stability of Xyl3088 was evaluated by studying the residual activities after incubating at 30 °C (Fig. 6b). Compared with the free Xyl3088, the storage stability of the immobilized one was sharply increased. After three-day storage, the free Xyl3088 only remained 21% of its initial activity, while the immobilized Xyl3088 preserved 62% of its initial activity. This indicated that silica NPs could afford suitable microenvironment and impose the steric constraints to the β-1,3-xylanase’s structure, preventing rapid denaturation. The improvement of thermostability was also suggestive of firm enzyme–support interactions, which perhaps arise from the enzyme entrapment that was possible owe to the one-step mild immobilization [31, 48].

**Table 1:** Half-life of the free and immobilized β-1,3-xylanase at different temperature

Temperature (°C)	Free β-1,3-xylanase	Immobilized β-1,3-xylanase
40	106.04 ± 10.75	113.23 ± 11.82***
45	20.44 ± 1.98	31.08 ± 2.06*
50	17.28 ± 0.46	28.06 ± 1.25**

t-test was performed on half-life of the free and immobilized β-1,3-xylanase at different temperatures. * indicate *p *< 0.05, **indicate *p *< 0.01 and ***indicate *p *< 0.001

**Fig. 7:**
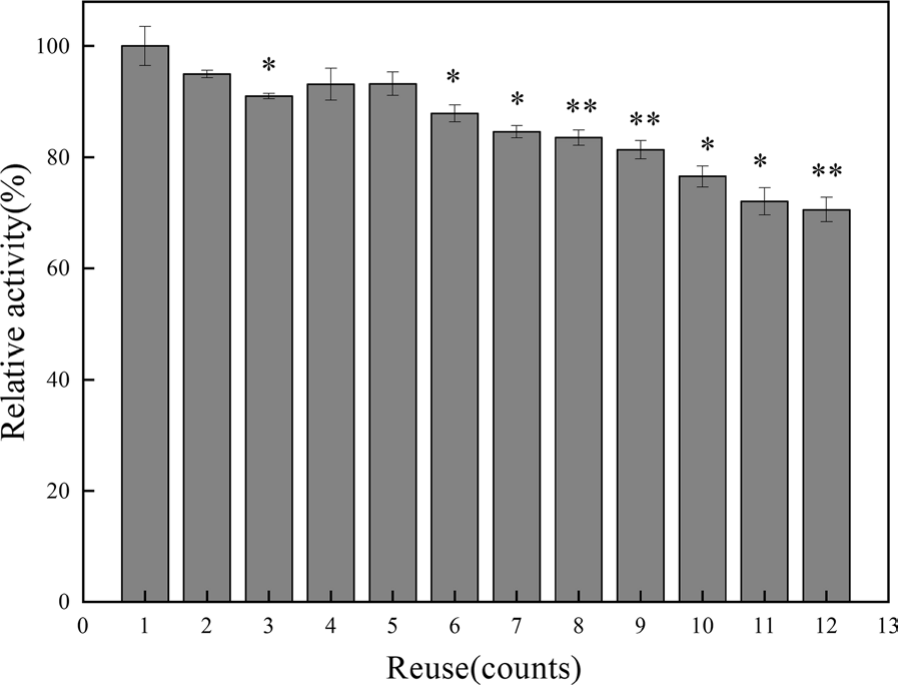
Reusability of the immobilized β-1,3-xylanases (Xyl3088) in PBS (pH 7.0) at 45 °C for 12 cycles. Error bars: the standard deviation of triplicate assays. * stands for significant difference with the control (the first run) (*p *< 0.05), ** stands for extreme significance (*p *< 0.01). Data are means ± standard errors

The main advantage of immobilized enzyme was the reuse potential, which will save the cost of the enzyme. β-1,3-xylanases immobilized on silica NPs retained 93.2% of its initial enzymatic activity after five cycles of successive reusing. Even after twelve cycles, the enzyme maintained around 70.6% of its initial activity (Fig. 7). The slight, but gradual decrease of remanent activity after each cycle may be attributed to several mechanisms, including the incidental loss of silica NPs during centrifugation and transfer in each cycle, and enzyme denaturation or structural modification of β-1,3-xylanases [32, 41, 49].

### Kinetic parameters of the free and immobilized β-1,3-xylanase

The Michaelis–Menten parameters (*K*m) and the catalytic efficiency (*K*cat/*K*m) of the free and immobilized β-1,3-xylanases (Xyl3088) were calculated. A slight increase in the *K*m was seen from the immobilized β-1,3-xylanases compared with the free ones (Table 2), indicating that the decreased affinity between enzyme and substrate. This was due to the conformational changes of β-1,3-xylanases by the immobilization carrier or a less accessibility of the active site of immobilized Xyl3088 to the substrate, especially the substrate was macromolecule β-1,3-xylan. Besides, the catalytic efficiency (*K*cat/*K*m) decreased from 113.05 mg/mL/s for the free Xyl3088 to 73.77 mg/mL/s for the immobilized Xyl3088 (*p *< 0.05). These results were consistent with most studies about enzyme immobilization. The decrease of active sites and the increase of mass transfer barriers may be responsible for the lower catalytic efficiency [50].

**Table 2:** The kinetic parameters of the free and immobilized β-1,3-xylanase

	*K*m (mg/mL)	*K*cat (s^−1^)	*K*cat/*K*m (mg/mL/s)
Free Xyl3088	5.23 ± 0.37	589.11 ± 4.39	113.05 ± 8.72
Immobilized Xyl3088	5.73 ± 0.45	513.15 ± 1.43**	73.77 ± 5.53*

* Stands for significant difference with the free Xyl3088 (*p *< 0.05), ** stands for extreme significance (*p *< 0.01). Data are means ± standard errors

Finally, the hydrolysates of the free and immobilized Xyl3088 were investigated by thin layer chromatography (TLC) to estimate their catalytic activities. As shown in Additional file 1: Fig. S2, the amount and type of hydrolysates differ little, including xylose (X1), xylobiose (X2), xylotriose (X3), xylotetraose (X4), and other oligosaccharides [2, 7].While the ion chromatography (IC) of hydrolysate produced by the immobilized Xyl3088 further confirmed the results of TLC (Additional file 1: Fig. S3). This revealed that immobilized carrier did not affect the catalytic process and hydrolysis products of β-1,3-xylanase. Where in the immobilized Xyl3088 could be recycled to hydrolyze the β-1,3-xylan from the seaweed to produce xylose. It also helps develop cheaper and eco-friendly biorefinery processes that convert xylose into valuable chemicals such as 2,3-butanediol, furfural and xylitol.

## Materials and methods

### Protein expression and purification

β-1,3-xylanase from the *Flammeovirga pacifica* strain WPAGA1 (Xyl3088, NCBI ID: MK253053), Xyl3088-SpyTag (X-T, NCBI ID: MN136290), K5V4F-SpyCatcher (K5-C, NCBI ID: MN136291) were all preserved in our lab and expressed in *E.coli* BL21(DE3), respectively. The protein expression was induced by 0.5 mM isopropyl-β-thiogalactopyranoside (IPTG) when OD600 was between 0.5 and 0.6. After shaking at 18 °C with 180 rpm overnight, the cells were harvested and disrupted by ultrasonication. The sonicated cell suspensions were all centrifuged at 4 °C for 20 min at 13,400×*g* to remove the insoluble cell debris. Then the cell lysate of Xyl3088 were filtered using a 0.45 μm sterile filter and then loaded into the nickel affinity column. Finally, the Xyl3088 were eluted using elution buffer (50 mM Tris–HCl buffer, 250 mM imidazole, 500 mM NaCl respectively, pH 7.0). Moreover, the cell lysate of K5-C protein was purified by the method of Inverse transition cycling (ITC). Briefly, 2.5 mol/L NaCl crystalline was loaded to the solution of K5-C which was kept in hot bath for 15 min at 37 °C to trigger K5-C phase transition, and the aggregated K5-C was harvested by centrifugation (12,000×*g*, 15 min) at 37 °C. Then the isolated K5-C was resuspended and kept in ice-cool PBS buffer for 1 h, then it was centrifuged at 4 °C (12,000×*g*, 10 min) to remove heteroprotein precipitation. The ITC process were repeated two times to purity the K5-C proteins [27, 51].

### Preparation of glycol β-1,3-xylan and β-1,3-xylooligosaccharides

Due to the β-1,3-xylan can’t obtain commercially, it was prepared from *Caulerpa lentillifera* (Nha trang, Vietnam) according to Iriki’s method [52], the insoluble β-1,3-xylan was then hydroxylated to form glycol β-1,3-xylan using Yamaura’s method [53]. The β-1,3-xylooligosaccharides were produced as follows, 0.1 g of β-1,3-xylan were dissolved in 10 mL of Tris–Cl buffer for 2 h under magnetic stirring at 180 rpm. Then 50 μL of free or immobilized β-1,3-xylanase was incubated with 350 μL of β-1,3-xylan suspension(1%, w/v)at 45 °C for 4 h. To terminate the enzymatic reaction, the solution was heated at 100 °C for 5 min, followed by centrifugation at 11,000*g* for 5 min. The supernatant containing the β-1,3-xylooligosaccharides was used for ion chromatography (IC) and thin layer chromatography (TLC) analysis.

The hydrolysate standard of β-1,3-xylan for thin-layer chromatography (TLC) analysis was prepared as following [54]: 0.5 g of β-1,3-xylan was suspended in 10 mL of 1 mol/L trifluoroacetic acid (TFA) solution and heated at 70 °C for 3 h. Then the solution was centrifuged to remove the insoluble β-1,3-xylan. Finally, the supernatant was neutralized with a 1 mol/L NaOH solution.

The first chromatographic separation was performed by an ion chromatography system (Dionex-ICS3000, American) equipping with a Dionex CarboPacPA-100 column (250 mm × 4 mm). The binary eluent was set to a flow rate of 300 µL/min using the gradient concentration of 200 mM NaOAc (100 mM NaOH). The column compartment was maintained at 30 °C throughout the run [55].

### Preparation of the self-assembled silica nanoparticles

The solution of orthosilicic acid was freshly prepared by loading 1.522 g tetramethyl orthosillicate (TMOS) to 10 mL hydrochloric acid (1 mM) and kept at room temperature for 10 min [56]. The orthosilicic acid and K5-C (300 μM) were fully mixed at 1:9 (v/v) ratio for 5 min. The mixture was centrifugated at 4 °C (5000*g*, 3 min), and the supernatant was gathered for quantifying the unencapsulated K5-C. The new formed silica nanoparticles (NPs) containing K5-C protein were washed three times with PBS buffer to remove excess silicic acid. Then the K5-C@SiO_2_ NPs were resuspended in 20 mM PBS buffer to observe the leakage of K5-C protein to the supernatant within 56 h. Meanwhile, the K5-C@SiO_2_ NPs were resuspended in deionized water and the suspension was loaded to copper grids to do the TEM analysis. At the same time, the K5-C@SiO_2_ NPs were also dried overnight at 60 °C to do the SEM and EDS analysis. Finally, the silica quantification was carried out by dissolving the K5-C@SiO_2_ NPs in 2 mol/L NaOH for 1 h at 37 °C. The amount of silica was measured at a wavelength of 370 nm using the β-silicomolybdate method [57, 58].

### One-step purification and immobilization of β-1,3-xylanase from cell lysate

1 mL cell lysate of β-1,3-xylanase was incubated with K5-C@SiO_2_ NPs in a tube tumbler at 37 °C for 1 h. As SpyTag and SpyCatcher could spontaneous form covalent bond in mild conditions, the silica NPs containing immobilized enzyme (K5-CT-X) were centrifuged and the supernatant was stored to quantify the immobilized and purified effect by SDS-PAGE. Meanwhile, the activity of the immobilized enzyme in PBS buffer were determined by the assay of the modified 3,5-dinitrosalicylic acid (DNS). The activity recovery and immobilization efficiency were calculated as following [59]:

$${\text{Activity recovery }}\left( \% \right) \, = \frac{{{\text{Observed activity }}\left( {\text{U}} \right)}}{{{\text{Starting activity of free enzyme }}\left( {\text{U}} \right)}} \times 100$$

$${\text{Immobilization efficiency }}\left( \% \right) \, = \, \frac{{{\text{Observed activity }}\left( {\text{U}} \right)}}{{{\text{Immobilized activity }}\left( {\text{U}} \right)}} \times 100$$

### The thermal stability, storage stability and reusability of β-1,3-xylanases

For thermal stability studies, the free and immobilized Xyl3088 were incubated in PBS buffer at 40 °C, 45 °C and 50 °C, respectively. The activities at 0 min were defined as 100%, residual activity was calculated as the percentage of initial activity at different periods (0, 5, 10, 15, 20 and 25 min). The half-life (t1/2) of enzyme was calculated according to Pinheiro’ method [60]. Storage stability of the free and immobilized Xyl3088 were measured by storing them in 50 mM Tris–HCl buffer (pH 7.0) at 30 °C for 72 h. The initial activity of Xyl3088 and immobilized one were assumed as 100%, while the residual activity was investigated at each time interval.

The reusability of immobilized Xyl3088 was evaluated in a 12-cycles repeated batch experiment (45 °C and pH 7.0). After each cycle, the silica NPs containing the immobilized Xyl3088 was harvested and resuspended in the fresh β-1,3-xylan solution to start a new cycle. The activity of the first cycle was defined as 100%. All experiments were carried out in triplicate.

### Kinetic parameters determination

Kinetic parameters of free or immobilized Xyl3088 were determined with increasing β-1,3-xylan concentrations from 1 mg/mL to 10 mg/mL by monitoring the concentration of reducing sugar at 540 nm. The Michaelis constant (Km) and maximum rate (Vmax) for free or immobilized Xyl3088 were calculated by the Michaelis–Menten model.

## Conclusions

Traditional immobilization strategies need multiple steps (for example: the carrier synthesis, activation and functional modification), which in some cases may be time-consuming and tedious. Besides, enzyme purification is usually required before immobilization, which may enhance the final economic cost of the immobilized enzymes. So, a novel all-in-one strategy for purification and immobilization of the β-1,3-xylanases was proposed, the target enzymes were immobilized via covalent bond by direct treatment of SpyCatcher modified silica nanoparticles with the cell lysate of SpyTag-xylanases. The strategy of immobilization was efficient compared with the traditional methods due to its mild conditions, simplicity, all-in-one step procedure and high enzyme loading efficiency. Furthermore, the immobilized β-1,3-xylanases displayed negligible leaching (0.5%), improved thermal stability, excellent reuse potential and good storage properties in respect to the free form, which will have great potentials in algae biomass treatment. Considering the critical factor using large or insoluble substrates for immobilized enzymes, the strategy we proposed would pave a new way for efficient enzyme immobilization.

## Supplementary information

**Additional file 1. Fig. S1** The flow chart of the all-in-one strategy for purification and immobilization of β-1,3-xylanases.** Fig. S2** Hydrolysis of β-1, 3-xylan by the free and immobilized β-1, 3-xylanases (Xyl3088). Lane M, molecular mass markers; lane 1, immobilized Xyl3088; lane 2, free Xyl3088.** Fig. S3** Product profiles of the hydrolysis reactions of β-1, 3-xylan by immobilized Xyl3088.** Table S1** Silica precipitating ability of various silica-mineralizing peptides.

## Data Availability

All data generated or analyzed during this study are included in this published article.

## Abbreviations

ELPs: Elastin-like polypeptide
PBS: Phosphate-buffered saline
ITC: Inverse transition cycling
K5-C: K5V4F-SpyCatcher
NPs: Nanoparticles
X-T: Xyl3088-SpyTag
