# Supplementary material for: A novel all-in-one strategy for purification and immobilization of β-1,3-xylanase directly from cell lysate as active and recyclable nanobiocatalyst
Source: Microb Cell Fact. 2021 Feb 6;20:37. doi: 10.1186/s12934-021-01530-5 (PMC7866670; doi:10.1186/s12934-021-01530-5)
Supplement: Supplementary file 1 — Additional file 1. Fig. S1 The flow chart of the all-in-one strategy for purification and immobilization of β-1,3-xylanases. Fig. S2 Hydrolysis of β-1, 3-xylan by the free and immobilized β-1, 3-xylanases (Xyl3088). Lane M, molecular mass markers; lane 1, immobilized Xyl3088; lane 2, free Xyl3088. Fig. S3 Product profiles of the hydrolysis reactions of β-1, 3-xylan by immobilized Xyl3088. Table S1 Silica precipitating ability of various silica-mineralizing peptides. [file 12934_2021_1530_MOESM1_ESM.docx]

Supplementary Materials

**A novel all-in-one strategy for purification and immobilization of β-1,3-xylanase directly from cell lysate as active and recyclable nanobiocatalyst**

Lixi Cai, Yunmen Chu, Xin Liu, Yue Qiu, Zhongqi Ge, Guangya Zhang*

**
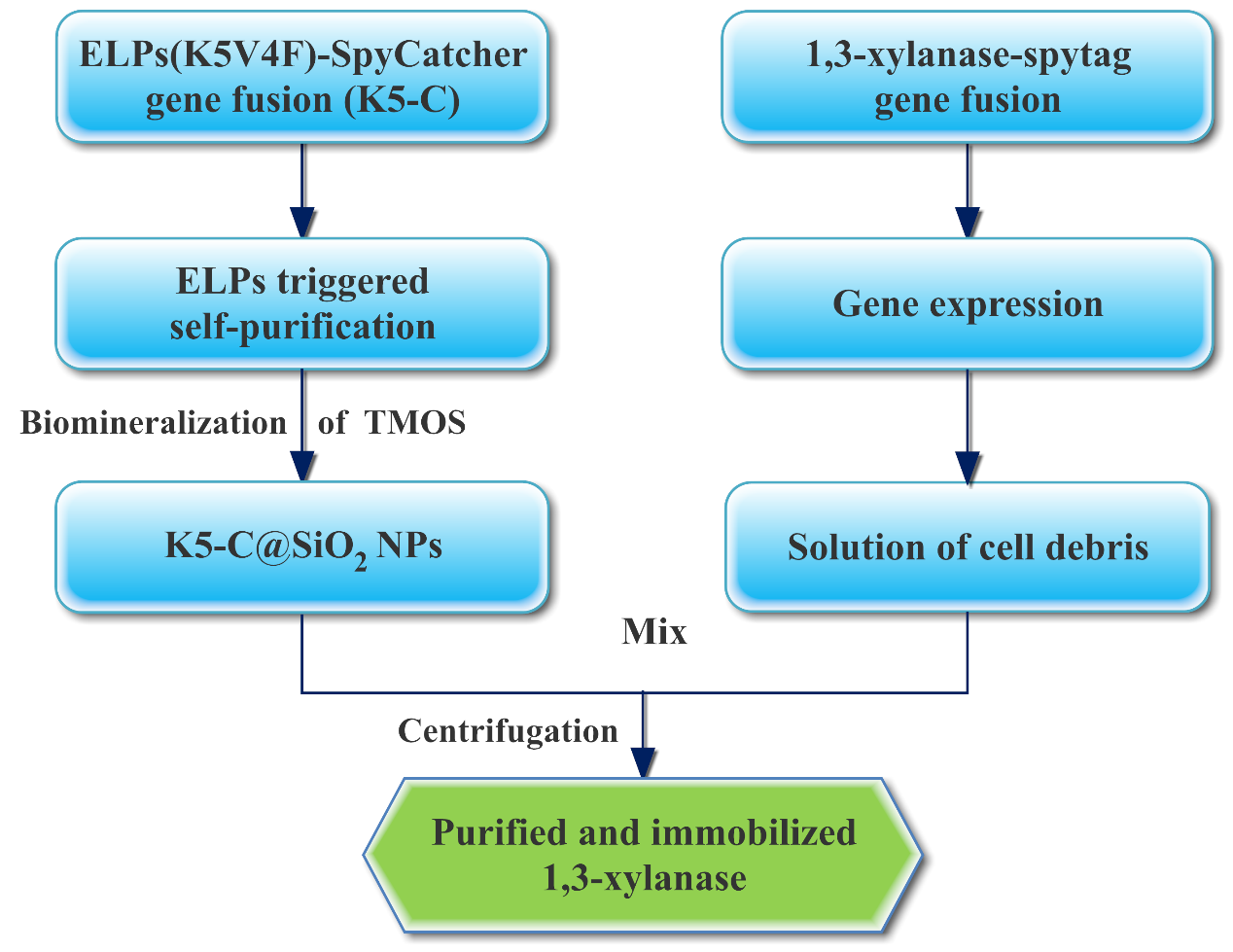
**

Fig. S1 The flow chart of the all-in-one strategy for purification and immobilization of β-1,3-xylanases


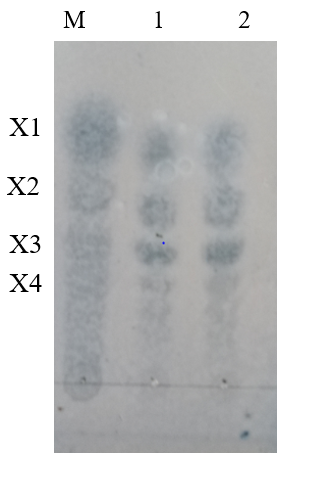


Fig. S2 Hydrolysis of β-1, 3-xylan by the free and immobilized β-1, 3-xylanases (Xyl3088). Lane M, molecular mass markers; lane 1, immobilized Xyl3088; lane 2, free Xyl3088.


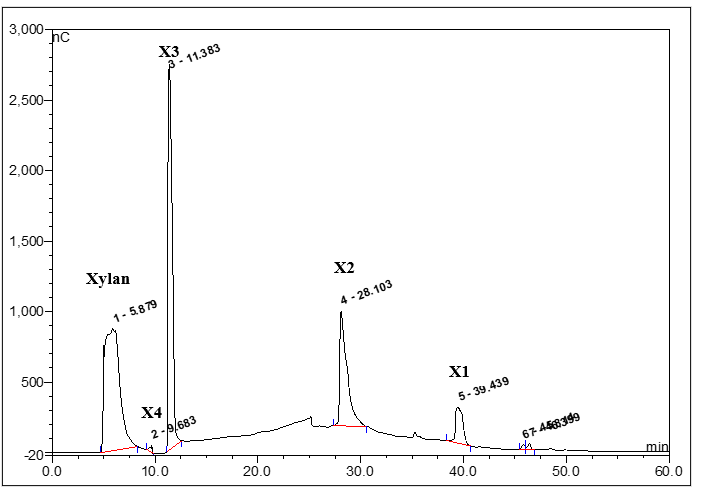


Fig.S3 Product profiles of the hydrolysis reactions of β-1, 3-xylan by immobilized Xyl3088.

**Table S1 Silica precipitating ability of various silica-mineralizing peptides.**

| Polypeptides | Product | Specific activity | Reference |
| --- | --- | --- | --- |
| R5 | White solid nanospheres | 3.59 ± 0.16 | 55 |
| Silaffin | White solid nanospheres | ~2.10 | 30 |
| ELP(KV8F) | White solid nanospheres | 97.18 ± 0.96 | 28 |
| K5-C(K5V4F-SpyCather) | White solid nanospheres | 99.93 ± 2.21 | This study |

Each value is the mean ± standard deviation (n = 3).
